# Alzheimer disease predicting from clinical and MRI data using DeepALZNET dual pathway framework

**DOI:** 10.1038/s41598-025-28221-0

**Published:** 2025-12-04

**Authors:** Saddam Bekhet, Nagwa Saad, Sara Farag

**Affiliations:** 1 Faculty of Commerce, South Valley University, Qena, 83523 Egypt; 2 Faculty of Computers and Artificial Intelligence, South Valley University, Qena, 83523 Egypt

**Keywords:** Alzheimer, MRI, Clinical data, Deep learning, Artificial intelligence, CNN, Random forest, Computational biology and bioinformatics, Image processing, Machine learning

## Abstract

Alzheimer’s Disease (AD) is a significant neurological condition characterized by progressive cognitive deterioration, with prevalence rising exponentially with age. Currently, there is no effective cure, and the disease progression impacts patients’ quality of life, often leading to severe symptoms before death. The gradual development of AD symptoms, often mistaken for typical aging, frequently leads to delayed diagnosis. This underscores the critical need for precise, early diagnostic methodologies, as timely intervention plays a crucial role in managing progression. Accordingly, this paper introduces DeepALZNET, a dual-pathway computational framework designed to enhance AD prediction by offering two independent processing pathways: one for structured clinical data and another for unstructured brain MRI scans. The first pathway combines a 1D Convolutional Neural Network (CNN) with a Random Forest classifier to analyze clinical data, while the second employs a transfer-learning-based VGG19 architecture to detect subtle structural changes in MRI scans. Empirical validation on two publicly available datasets (2k clinical cases and 40k MRI images) demonstrated that both pathways achieved competitive accuracy ($>90\%$), with further evaluation on ADNI and OASIS benchmarks confirming robustness. Unlike recent transformer-based or attention-driven methods, which often demand large multimodal datasets and high computational resources, DeepALZNET prioritizes practical applicability, interpretability, and adaptability, operating effectively with either clinical or imaging data alone. This design bridges the gap between benchmark-driven research and deployable real-world solutions, with potential for multimodal fusion or attention integration in future work.

## Introduction

Millions of people worldwide suffer from AD, a progressive and complex neurological illness that is the primary cause of dementia^1^. Like other forms of dementia, the risk increases with age, but it can also occur in midlife, between a person’s 30s and mid-60s^2^. AD is characterized by a progressive deterioration in cognitive abilities, such as memory loss, confusion, impaired decision-making, and challenges with language and reasoning, which progressively worsen. This decline makes it increasingly difficult for affected people to carry out daily activities, which places a significant burden on healthcare systems^3^. The increased occurrence of AD, especially in older adults, has made early identification crucial for improving patient outcomes^4,5^.

Traditional diagnostic methods, which mostly depend on clinical assessments and neuroimaging techniques like MRI scans, usually detect the illness in its later stages, after significant brain damage has occurred. These techniques can be time-consuming and sometimes fail to capture the subtle changes in brain structure that occur during the early stages of AD^6^. Furthermore, such approaches can be subjective. But progress in Artificial Intelligence (AI), especially in Machine Learning (ML) and Deep Learning (DL), has shown a lot of promise for finding AD earlier by analyzing big, complicated datasets^7^. AI-based systems can spot patterns linked to the early stages of AD. They can also process diverse datasets, such as clinical markers and demographic data, in addition to imaging data, to improve diagnostic precision. More accurate early-stage diagnoses, can be provided by ML techniques by analyzing various biomarkers.

In clinical settings, the type of data available for a patient might vary. Sometimes detailed clinical histories and assessment scores are the primary information, while other times recent MRI scans are the main resource. To address this practical variability, this paper introduces DeepALZNET , a dual-pathway deep learning framework for Alzheimer’s disease prediction. DeepALZNET is designed to operate effectively using either clinical data or brain MRI images independently. This modality-specific processing capability allows the framework to provide predictive analysis based on the available patient information.

The framework consists of two distinct pathways: The first pathway is tailored for structured clinical data, combining a CNN with a Random Forest classifier. The second pathway is designed for neuroimaging analysis, employing a deep CNN architecture (specifically, an enhanced VGG19 model utilizing transfer learning) to analyze 2D MRI scans. By supporting these distinct data types, DeepALZNET offers a flexible approach to computational AD assessment.

The remaining parts of this paper are ordered as follows: “Related works” summarizes related work. In “The proposed DeepALZNET framework”, the proposed DeepALZNET framework, detailing its two pathways, is presented. The experimental setup, datasets, results, and discussion are provided in “Experimental results and discussion”. Finally, concluding remarks and future work are discussed in “Conclusion”.

## Related works

The application of DL and ML techniques for early-stage AD diagnosis and prediction has been the core subject of numerous studies. The usefulness of ensemble learning strategies, like boosting and bagging, which integrate several classifiers to greatly enhance performance in comparison to single models, was emphasized by^8^. Their accuracy ratings varied from 80% to 86.92% based on Open Access Series of Imaging Studies (OASIS)^9,10^. This method makes use of a variety of clinical data sources, including the results of cognitive tests and neuroimaging, to produce predictions that are more accurate and robust.

With explainable artificial intelligence (XAI),^11^ seeks to offer model explanations that guarantee efficiency and build confidence. By using sub-scores from clinical evaluations and merging them with ADNI dataset^12^ sub-scores for more accurate AD prediction, the novel multi-level stacking model demonstrates an enhancement in early disease diagnosis. Additionally, the multi-level stacking models outperformed single-level stacking and classical machine learning models. Aiming to improve AD detection and reduce overfitting,^13^ presented a uni-data, multi-model framework. Their approach combined machine learning and 3D-ResNet models on the ADNI MRI dataset, with performance boosted by adding demographic and cognitive information.

The use of deep learning techniques in the analysis of medical images has increased, often enabling faster analysis and better accuracy than a human practitioner^14^. Various combinations of existing models (Ensemble learning techniques) can enhance the predictive accuracy of better-performing models. By leveraging the strengths of multiple models, ensemble learning can help produce more accurate and reliable results^14,15^. The application of DL models in clinical contexts has been significantly enhanced with the emergence of XAI frameworks. According to^14^, combining XAI with DL models improves the clinical usefulness of AI-driven diagnostic tools by assisting physicians in understanding the features, such as particular brain regions or biomarkers, that affect the predictions. Furthermore, hybrid models, which fuse deep learning and machine learning techniques, have shown to be especially successful in diagnosing AD. These hybrid models offer enhanced diagnostic robustness, accuracy, and generalization when processing large and diverse datasets, as^16^ and^15^ showed.

As highlighted by the existing literature, significant progress has been made in leveraging computational methods, particularly deep learning, for AD diagnosis using various data modalities. Researchers have explored unimodal approaches focusing on clinical data or MRI scans, as well as generic multimodal fusion techniques. However, a common limitation is the reliance on specific data types being available, or the requirement to fuse data from multiple sources for the same patient, which may not always be available in routine clinical practice. There is a need for flexible, robust diagnostic tools that can perform effectively even when only one modality of data is available, while still incorporating advanced learning techniques and providing insights into the basis of their predictions. Addressing this gap, this paper proposes DeepALZNET , a novel dual-pathway framework designed to offer high-accuracy AD prediction utilizing either clinical data or MRI scans independently, integrating advanced deep learning and machine learning methods tailored for each specific data type. A comparative summary of DeepALZNET and representative models from the reviewed literature, highlighting differences in data modalities, architectures, interpretability, fusion strategies, and datasets, is presented in Table 1.

**Table 1:** Comparative summary of DeepALZNET and representative models from the literature, highlighting modality, architecture, interpretability, fusion strategy and datasets.

Study	Data modality	Architecture	Interpretability	Fusion strategy	Dataset(s)
DeepALZNET (Ours)	Clinical + MRI	Pathway1: CNN+RF, Pathway2: VGG19	CAM, Feature Importance	Independent Dual Pathways	Kaggle Clinical/MRI, ADNI, OASIS
Adarsh et al. (2024)^17^	MRI + Clinical	CNN, 3D U-Net, VGGNet 3D	Grad-CAM	Late Fusion	OASIS, ADNI
Tanveer et al. (2020)^18^	MRI + Clinical	ResNet152, InceptionV3, Naive Bayes, AdaBoost	Grad-CAM	None	OASIS, ADNI
Saratxaga et al. (2021)^19^	MRI	BrainNet2D	None	N/A	OASIS
Almohimeed et al. (2023)^11^	Clinical	Ensemble (MLP + RF)	SHAP	N/A	ADNI
Kavitha et al. (2022)^8^	Clinical	Random Forest	None	N/A	OASIS
Mumuni et al. (2022)^20^	Clinical	ML Models + SMOTE	None	N/A	–
Bekhet et al. (2024)^21^	MRI	CNN + Genetic Algorithm Feature Selection	CAM	None	–
Sethi et al. (2022)^22^	MRI	CNN + SVM	None	N/A	OASIS
Shaffi et al. (2024)^23^	MRI	Naive Bayes	None	N/A	ADNI
Ke et al. (2017)^24^	Clinical	LightGBM (Feature Importance)	Feature Importance	N/A	–
Zhou et al. (2016)^25^	MRI	CAM Concept	CAM	N/A	–
Braak et al. (1991)^26^	Neuropathology	Neuropathological Staging	N/A	N/A	–

## The proposed DeepALZNET framework

This section presents the proposed DeepALZNET framework, a dual-pathway computational system designed for flexible and accurate Alzheimer’s disease prediction based on the type of input data available. Unlike conventional multimodal systems that require fusing different data types for the same patient, DeepALZNET offers two distinct and fully independent processing pathways: one optimized for structured clinical data and another for unstructured MRI brain images. This architecture enables the framework to adapt seamlessly to varying clinical scenarios, ensuring that prediction remains possible even when only one type of data is available.

While multimodal fusion strategies^27^ including early fusion at the feature level, late fusion at the decision level, and joint embedding methods have demonstrated strong performance in AD prediction, our approach deliberately prioritizes pathway independence. This decision is grounded in the practical realities of healthcare environments, where complete multimodal datasets for the same patient are often unavailable due to financial constraints, acquisition time, or patient condition^28^. By allowing each pathway to function autonomously, DeepALZNET maintains broad applicability and is more easily deployed in diverse contexts.

**Figure 1:**
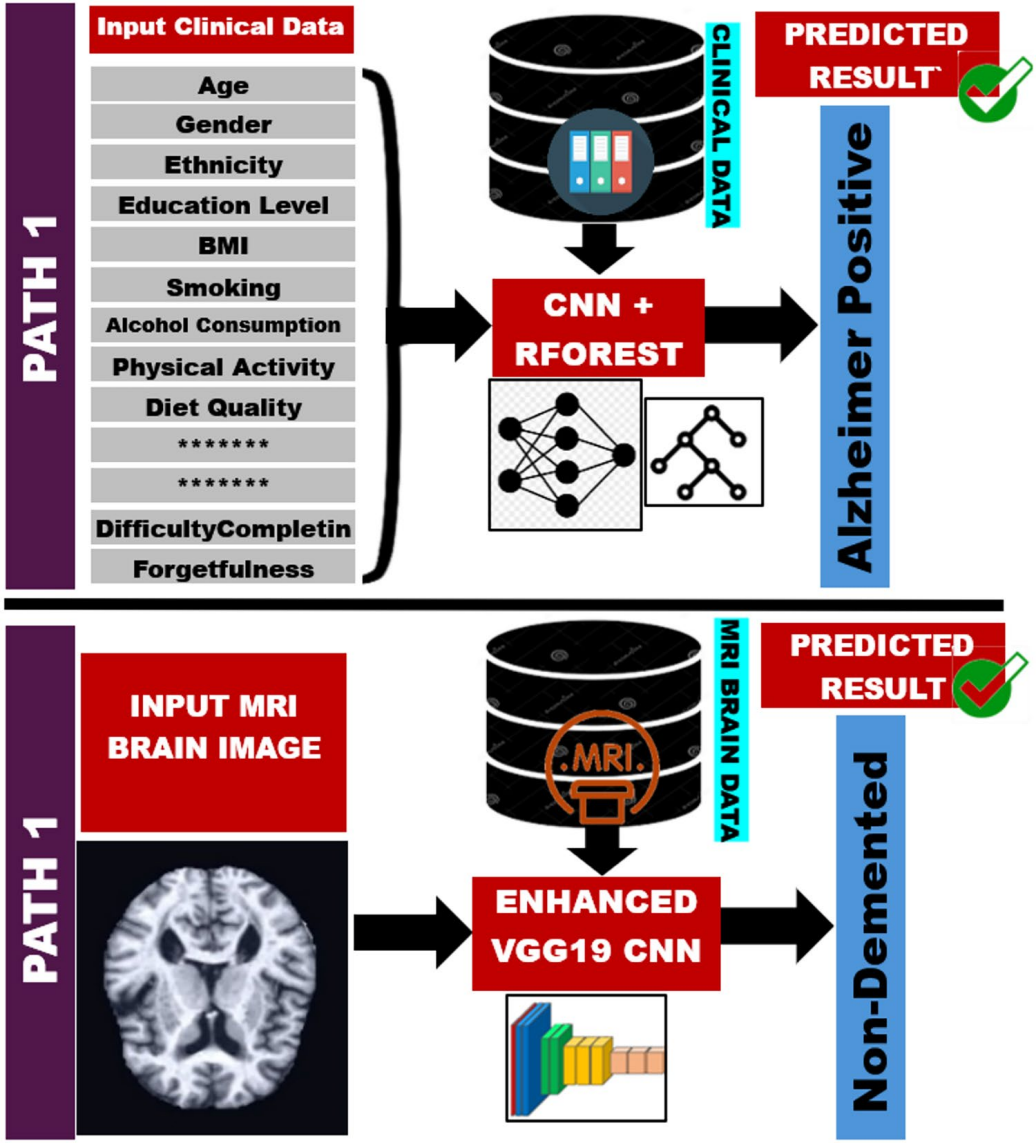
Abstract diagram of the proposed dual-pathway Alzheimer’s prediction framework (DeepALZNET), showcasing the two independent pathways for clinical data and MRI images.

Figure 1 provides a high-level visualization of this architecture. The framework routes the input to the corresponding pathway depending on the detected data type, as described in the following subsections.

### Pathway-1: clinical data analysis using CNN and random forest

When the input consists of structured clinical data for a patient, Pathway1 is activated. This pathway is designed to analyze potentially high-dimensional and heterogeneous clinical information, which can include demographic details, medical history, lifestyle factors, and cognitive assessments (as summarized in Table 3). The challenge here is to effectively capture complex interactions and patterns within this data without relying on standard manual feature engineering.

The clinical data for a patient is preprocessed and represented as a numerical feature vector $\textbf{x}_{clin} \in \mathbb {R}^D$, where *D* is the total number of features (e.g., after encoding categorical variables and normalization). This pathway employs a hybrid model combining a 1D Convolutional Neural Network (CNN) with a Random Forest (RForest) classifier.

A 1D CNN is applied to the clinical feature vector $\textbf{x}_{clin}$ to automatically learn abstract representations. While 1D CNNs are typically used for sequential data, here they are employed in an exploratory manner to potentially identify complex, localized interactions among features that might not be captured by traditional linear models or even standard feedforward networks. The convolutional layers apply filters across the feature vector, capturing dependencies within defined windows of features. The output of a convolutional layer *l*, $\textbf{h}_l$, is given by:

1 $$\begin{aligned} \textbf{h}_l = f(\textbf{W}_l * \textbf{h}_{l-1} + \textbf{b}_l) \end{aligned}$$

where $\textbf{h}_{l-1}$ is the input (initially $\textbf{x}_{clin}$), $*$ denotes the 1D convolution, $\textbf{W}_l$ and $\textbf{b}_l$ are learnable parameters, and *f* is the activation function. Pooling layers follow to downsample and increase robustness. The final layers of the 1D CNN produce a dense, learned feature representation vector $\textbf{h}_{cnn}$.

This extracted feature vector $\textbf{h}_{cnn}$ serves as the input to a Random Forest classifier. Random Forest is an ensemble learning method that constructs multiple decision trees (*T*) and combines their predictions, typically through majority voting for classification^29^. This method is chosen for its robustness to overfitting and ability to handle high-dimensional input. The RForest is trained on the $\textbf{h}_{cnn}$ vectors derived from the training data. The final prediction $\hat{y}_{clin}$ from Pathway1 is determined by the RForest classifier:

2 $$\begin{aligned} \hat{y}_{clin} = \text {majority}\_\text {vote}_{t=1}^{T} \{ \text {tree}_t(\textbf{h}_{cnn}) \} \end{aligned}$$

where $\text {tree}_t(\textbf{h}_{cnn})$ is the class prediction of the $t^{th}$ decision tree given the CNN-extracted features. This hybrid approach leverages the CNN’s ability to automatically learn feature interactions from the raw clinical vector and the RForest’s ensemble power for robust classification.

To assess the relative contributions of the CNN-based feature extractor and the Random Forest classifier in Pathway 1, we conducted an ablation study using the Kaggle Clinical dataset (introduced later). Three variants were tested: (a) the full hybrid 1D CNN + Random Forest architecture, (b) 1D CNN with a dense softmax classification head, and (c) Random Forest trained directly on preprocessed clinical features. As shown in Table 2, the hybrid configuration achieved the highest accuracy (90 %), outperforming the 1D CNN-only (88.7%) and Random Forest-only (86.5%) baselines. This confirms that CNN-based feature extraction captures non-linear dependencies and local interactions in the clinical feature space, while the Random Forest’s ensemble mechanism provides enhanced generalization and robustness.

**Table 2:** Ablation study results for Pathway 1 (Clinical Data) on the Kaggle dataset.

Configuration	Accuracy (%)	F1-score
1D CNN + Random Forest (Full Pathway 1)	**90**	**0.901**
1D CNN only	88.7	0.884
Random Forest only	86.5	0.861

### Pathway-2: MRI image analysis using a deep CNN

When the input is a 2D brain MRI scan, Pathway2 is activated. This pathway utilizes a deep convolutional neural network architecture, specifically a VGG19-based model^30^, for analyzing neuroimaging data to detect morphological changes potentially demonstrative of AD.

The input is a 2D MRI brain image, denoted as $I_{mri}$. Preprocessing steps, including resizing to a standard input size for the VGG19 model and normalization, are applied. To enhance training robustness and generalize better, data augmentation techniques (described in “Network training phase”) are used. The VGG19 model is employed, with weights pre-trained on the large ImageNet dataset^31^. This transfer learning approach initializes the network with a rich set of visual features that provide a strong starting point for medical image analysis. The VGG19 architecture consists of sequences of convolutional layers with small $3 \times 3$ kernels, followed by max-pooling layers, designed to capture increasingly complex spatial hierarchies.

The pre-trained VGG19 convolutional layers are typically fine-tuned on the target MRI dataset with a small learning rate, while the fully connected layers are trained from scratch or with a higher learning rate to adapt to the specific AD classification task (e.g., classifying into Non-Demented, Very Mild, Mild, Moderate Demented). The final layer is a dense layer with a softmax activation function to output class probabilities $p_k$:

3 $$\begin{aligned} p_k = \frac{e^{z_k}}{\sum _{j=1}^{C} e^{z_j}} \quad \text {for } k = 1, \dots , C \end{aligned}$$

where $z_k$ is the output score for class *k* from the preceding dense layer, *C* is the number of AD stages/classes, and $p_k$ is the predicted probability for class *k*. The final prediction $\hat{y}_{mri}$ from Pathway 2 is the class with the highest predicted probability:

4 $$\begin{aligned} \hat{y}_{mri} = \arg \max _{k} (p_k) \end{aligned}$$

This pathway utilizes the proven capability of deep CNNs and the benefits of transfer learning to directly classify AD stages based on structural patterns in MRI scans. The overall operational logic of the DeepALZNET framework, which selects the appropriate pathway based on the input data type, is summarized in Algorithm 1.

**Algorithm 1:**
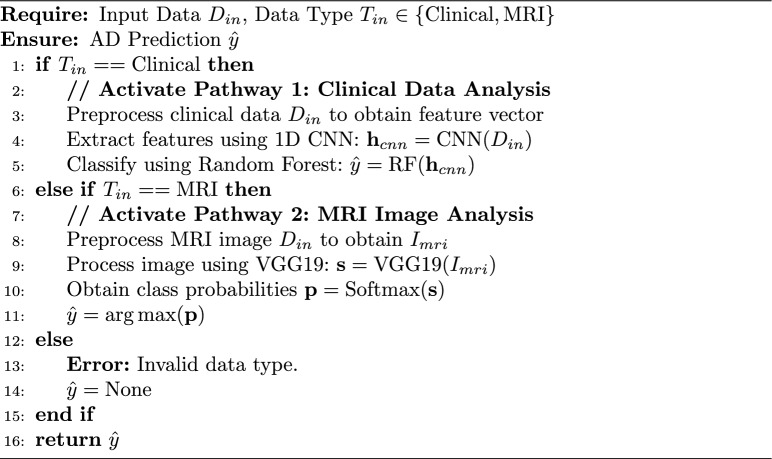
AD Prediction Algorithm

## Experimental results and discussion

The performance and effectiveness of the proposed DeepALZNET framework’s two independent pathways were evaluated through experiments conducted on distinct Alzheimer’s disease datasets: one comprising structured clinical data and the other containing brain MRI images. This section details the datasets used, the training process for each pathway, the metrics employed for quantitative assessment, and provides a comprehensive discussion of the achieved results. For comparison with existing literature, preliminary evaluations were also performed on subsets of the ADNI and OASIS datasets. The specific details of these subsets and experimental setups are discussed in the relevant result subsections.

### Datasets

**Figure 2:**
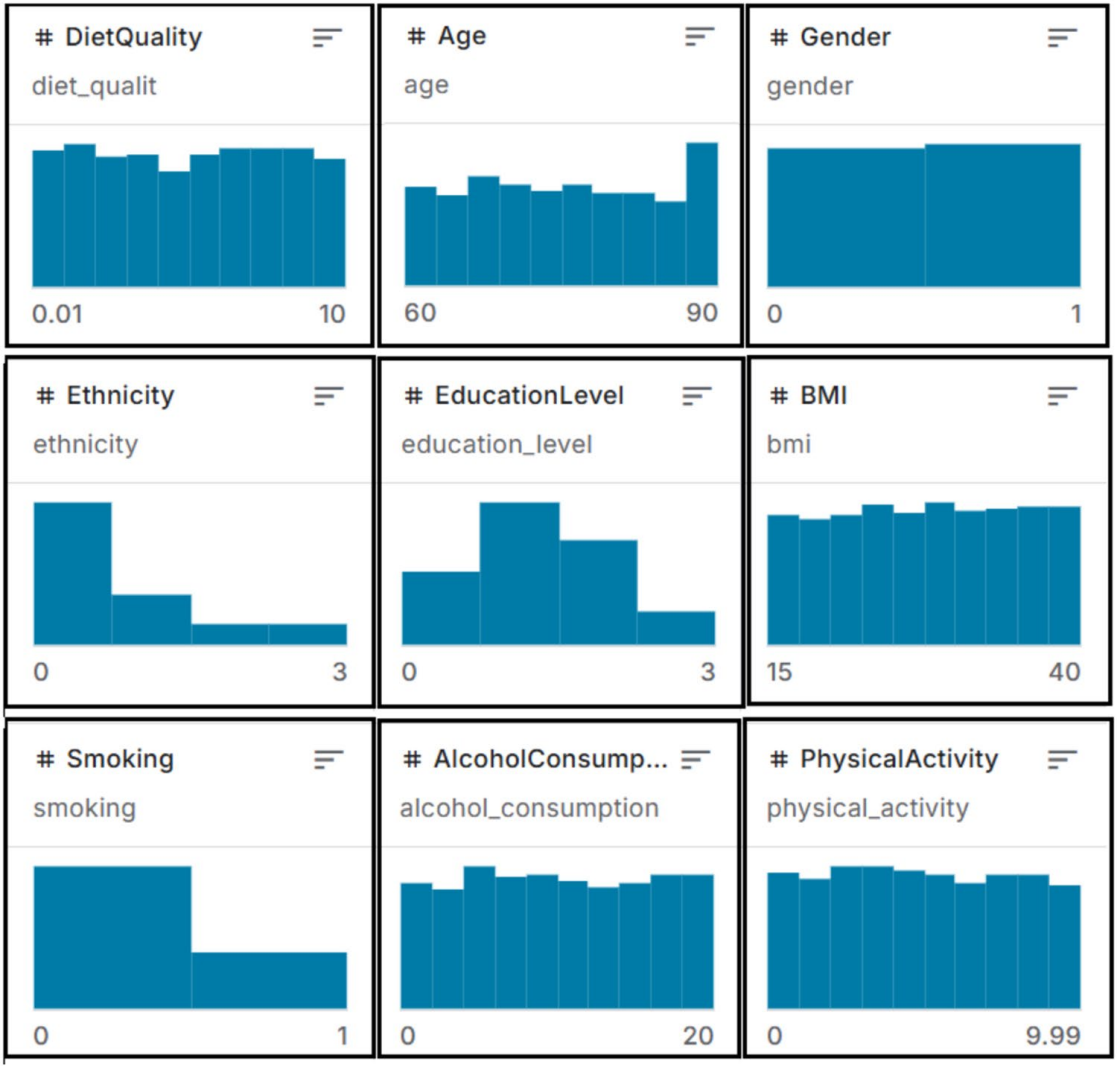
Visualization of the features’ range of values of the Alzheimer’s clinical dataset^32^.

The proposed framework operates on two types of data: clinical data and brain MRI images. The first dataset is a publicly available clinical dataset obtained from Kaggle^32^, containing 2k patient records. This dataset includes a range of structured information such as demographic details, lifestyle factors, medical history, clinical measurements, cognitive and functional assessments, and symptom reports, with a final diagnosis of Alzheimer’s Disease. The dataset comprises the attributes described in Table 3, including detailed sub-features within the listed categories. The range of values for these features is visualized in Fig. 2.

**Table 3:** Overview of feature categories in the Alzheimer’s clinical dataset^32^.

Feature category	Description (selected examples)
Demographic details	Age, Gender
Ethnicity	Patient ethnicity
Education level	Patient education level
Lifestyle factors	BMI, smoking status, alcohol consumption, physical activity, diet quality, sleep quality
Medical history	Family history of AD, cardiovascular disease, diabetes, depression, head injury, hypertension
Clinical measurements	Systolic BP, diastolic BP, total cholesterol, LDL, HDL, triglycerides
Cognitive and functional assessments	MMSE score, functional assessment score, memory complaints, behavioral problems, ADL score
Symptoms	Confusion, disorientation, personality changes, difficulty completing tasks, forgetfulness
Diagnosis information	Diagnosis status for Alzheimer’s disease (binary: yes/no)

The dataset includes specific features within each category.

**Figure 3:**
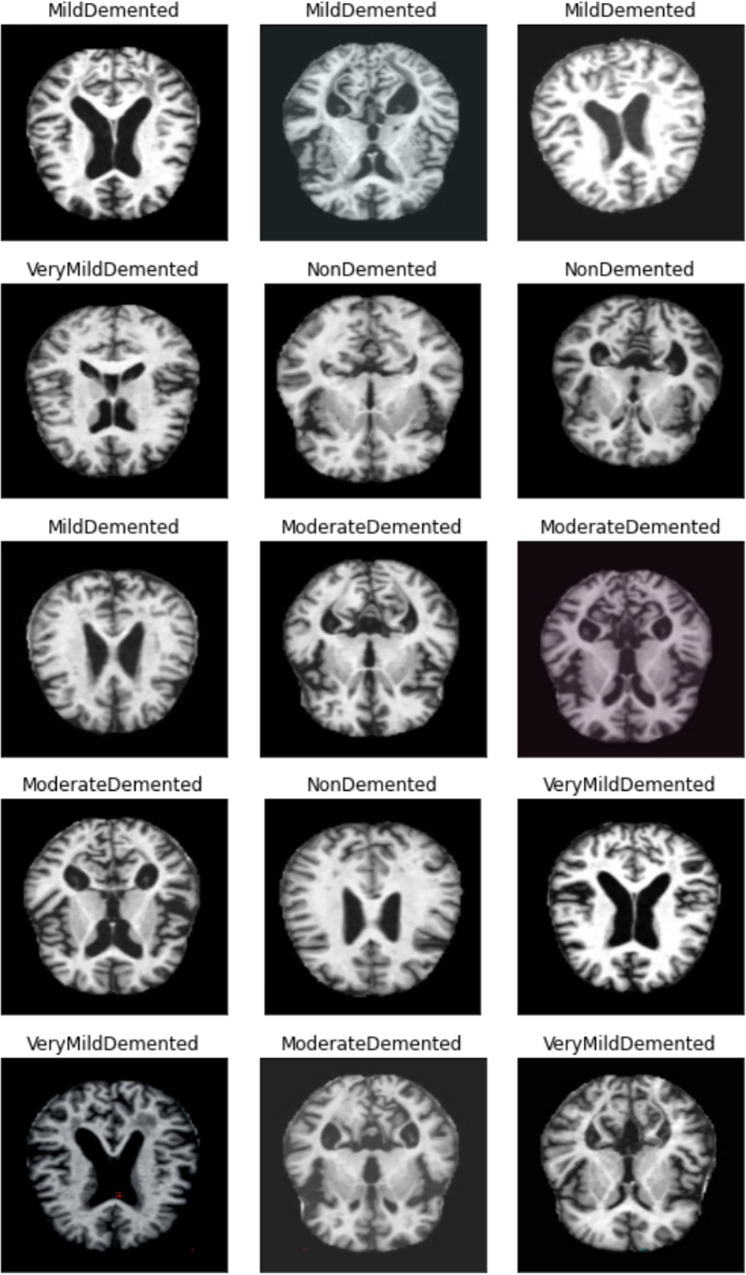
Sample images from the Alzheimer’s MRI brain dataset^33^ for each of the four classes.

The second dataset consists of 40k 2D brain MRI images obtained from Kaggle^33^. This dataset is intended for the classification of Alzheimer’s disease severity and contains images categorized into four classes: Mild Demented, Moderate Demented, Non Demented, and Very Mild Demented. The dataset was prepared by extracting 2D slices from 3D MRI scans and includes axial, sagittal, and coronal views, though specific slice locations are not provided in the dataset documentation. Figure 3 depicts sample images from each class. The distribution of samples across classes in the MRI dataset is as follows: Mild Demented (approx. 6.5k), Moderate Demented (approx. 0.5k), Non Demented (approx. 22.4k), and Very Mild Demented (approx. 10.6k). Both datasets (clinical and MRI) were accessed under public licenses.

### Network training phase

The training for both framework pathways was conducted separately using their respective datasets. Experiments were performed on a system equipped with an Intel Core i7 CPU and an NVIDIA GPU (e.g., NVIDIA RTX 3060). The following subsections detail the training processes for each data type on the primary Kaggle datasets. Details regarding the evaluations on ADNI and OASIS datasets are provided in the results section where comparisons are discussed.

#### Pathway1: clinical data based training

The training process for Pathway1 begins with comprehensive preprocessing of the clinical data. This includes handling any missing values by imputation (e.g., mean or median imputation), encoding categorical features (e.g., One-Hot Encoding), and standardizing numerical attributes to have zero mean and unit variance. To mitigate the impact of class imbalance between the ”Alzheimer’s” and ”No Alzheimer’s” classes (which is typical in disease datasets), techniques such as SMOTE^20^ (Synthetic Minority Over-sampling Technique) were applied to the training data.

The preprocessed clinical data feature vectors were used to train the 1D CNN. This network was configured with convolutional and pooling layers designed to extract abstract features from the input vector. For our experiments on the Kaggle clinical dataset, this CNN was trained for 50 epochs with a batch size of 32 using the Adam optimizer. Binary cross-entropy loss was used, along with weight decay of 0.0001, and class weighting to improve robustness and handle class distributions.

Following the training of the 1D CNN, the learned feature representations from the penultimate dense layer were extracted for all samples (training and testing). These extracted features ($\textbf{h}_{cnn}$) served as input to the Random Forest classifier. The Random Forest model, consisting of 100 decision trees, was then trained on these CNN-derived features using the original class labels. The final prediction from Pathway1 on this dataset is the output of this trained Random Forest model.

#### Pathway2: MRI data based training

For Pathway2, the MRI brain images from the Kaggle dataset were preprocessed by resizing to a standard input size (i.e., 224x224 pixels) suitable for the VGG19 model and normalizing pixel intensity values. To combat overfitting and improve generalization, data augmentation^21^ was applied on-the-fly during training using Keras’s ImageDataGenerator. This included random translations (up to 15% of width/height), random rotations (up to 15 degrees), random horizontal reflections, and random zoom (up to 15%).

The VGG19-based model, pre-trained on ImageNet^34^, was used. The convolutional base layers were loaded with pre-trained weights. A new classification head consisting of fully connected layers was added on top. The model utilized the Adam optimizer for training. Categorical cross-entropy was used as the loss function for the four-class classification problem. Training parameters included a batch size of 10 examples, a learning rate of $1e^{-4}$, and a weight decay of 0.0004. Leveraging transfer learning, the initial layers of the VGG19 convolutional base were initially frozen and only the new classification head was trained. Subsequently, these layers were fine-tuned with a smaller learning rate of $1e^{-5}$, along with the rest of the network. Training proceeded for 50 epochs, monitoring the validation loss for convergence.

### Results and discussion

For evaluating the performance of both pathways on the Kaggle datasets, each dataset was randomly split into a training set (70%) and a test set (30%).. The split was performed once for each dataset. Quantitative evaluation utilized standard classification metrics: Accuracy (Eq. (5)) and F1-score (Eq. (6)). The validation loss (Categorical Crossentropy for Pathway2, Binary Crossentropy for Pathway1) as defined in Eq. (7) were used as well to assess model generalization.

5 $$\begin{aligned} \text {Accuracy}= & \frac{\text {Number of Correct Predictions}}{\text {Total Number of Samples}} \end{aligned}$$

6 $$\begin{aligned} F1= & \frac{2 \times \text {Precision} \times \text {Recall}}{\text {Precision}+\text {Recall}} \end{aligned}$$

7 $$\begin{aligned} J= & \frac{1}{N}\sum \limits _{i=1}^{N} \mathcal {L}(\hat{y}_i,y_i) \end{aligned}$$

where *N* is the number of samples, $y_i$ is the true label, $\hat{y}_i$ is the predicted label, and $\mathcal {L}$ is the loss function (Binary or Categorical Crossentropy).

#### Pathway1: clinical data results

**Figure 4:**
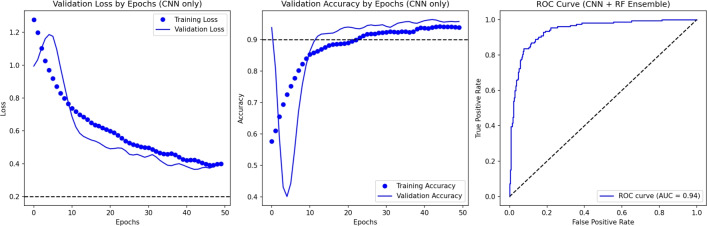
Training and validation loss and accuracy curves for the CNN component of Pathway1 over 50 epochs, alongside the ROC curve for the complete hybrid model (CNN + Random Forest). The CNN curves demonstrate stable convergence, while the ROC highlights the strong discriminative ability of DeepALZNET Pathway1.

The DeepALZNET Pathway1 model (1D CNN + RForest) achieved an overall accuracy of 90.2% on the clinical test dataset from Kaggle. Figure 4 illustrates the training and validation loss and accuracy curves for the CNN component of the pathway, confirming stable convergence and consistent generalization. The final validation loss stabilized at 0.35. In addition, the ROC curve shown in the same figure represents the performance of the complete hybrid Pathway1 (CNN + Random Forest), achieving a strong discriminative capability with an AUC of 0.95. The detailed classification report provides per-class metrics for the binary classification task:

- Class 0 (No Alzheimer’s): F1-score of 92%
- Class 1 (Alzheimer’s): F1-score of 86%

These results indicate that the model effectively learned discriminatory patterns from the structured clinical features. The combination of the CNN’s feature extraction and the RForest’s robust classification contributed to achieving this high accuracy on the clinical data. The confusion matrix for the clinical model on this dataset is shown in Fig. 6, illustrating the counts of correct and incorrect predictions for each class.

#### Pathway 2: MRI data results (Kaggle dataset)

**Figure 5:**
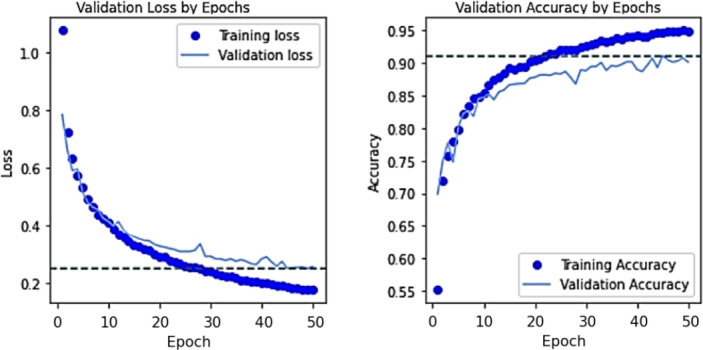
Training progress of DeepALZNET Pathway2 (VGG19-based) on the Kaggle MRI dataset over 50 epochs, showing accuracy and loss curves.

The DeepALZNET Pathway2 model (VGG19-based) achieved an overall accuracy of 92.2% on the Kaggle MRI brain image test dataset. Figure 5 displays the training progress, showing the convergence of accuracy and loss. The final validation loss was approximately 0.28. The F1-scores for each class on the test dataset were:

- Mild Demented: 0.93
- Moderate Demented: 1.00
- Non Demented: 0.95
- Very Mild Demented: 0.92

**Figure 6:**
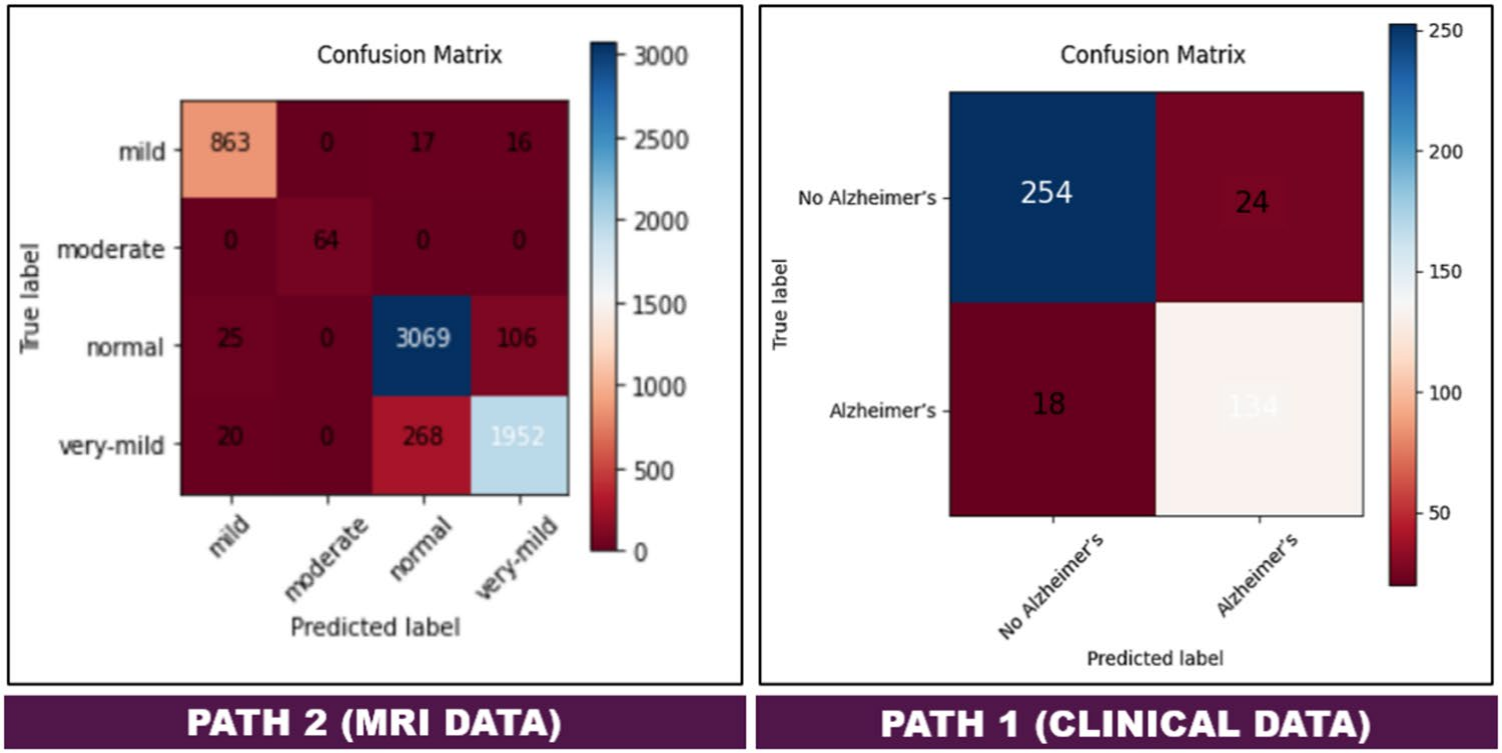
Confusion matrices for (left) DeepALZNET Pathway1 on the Kaggle clinical dataset and (right) DeepALZNET Pathway 2 on the Kaggle MRI dataset.

The F1-score of 1.00 for the Moderate Demented class indicates perfect classification for this specific category in the test set. Given the small number of samples in this class in the full dataset (approx. 0.5k), this perfect score on the 30% test split should be interpreted with caution, as it might be due to favorable sampling in this particular split rather than guaranteed perfect performance on all unseen data. The confusion matrix for the MRI model (Fig. 6 (right)) shows that while overall accuracy is high, there is some notable confusion between ”Non Demented” and ”Very Mild Demented” cases, and between ”Very Mild Demented” and ”Mild Demented” cases, reflecting the subtle differences between these stages and the inherent difficulty in classifying them based solely on image morphology.

To address the inherent class imbalance in the MRI dataset^33^ particularly the underrepresentation of the Moderate Demented class we explored advanced imbalance-aware strategies beyond the SMOTE^20^ and undersampling methods used in the initial setup. In addition to standard categorical cross-entropy, we evaluated focal loss^35^, which dynamically down-weights the loss contribution of well-classified examples, thereby focusing learning on harder, minority-class samples. This was complemented by a discussion of class-balanced loss^36^ and sample weighting approaches, which adjust gradient updates according to the effective number of samples per class. A supplementary experiment using focal loss ($\gamma$ = 2.0) in Pathway2 resulted in a stable overall accuracy (91.5% vs. 92.2% baseline) but yielded a noticeable improvement in the Moderate Demented class F1-score (0.82 vs. 0.76), along with an increase in the macro F1-score (0.902 vs. 0.884). These results confirm that targeted loss reweighting can improve minority-class recognition without sacrificing global accuracy, and such strategies remain promising for future refinements of DeepALZNET .

### Key clinical data in Alzheimer’s classification

**Figure 7:**
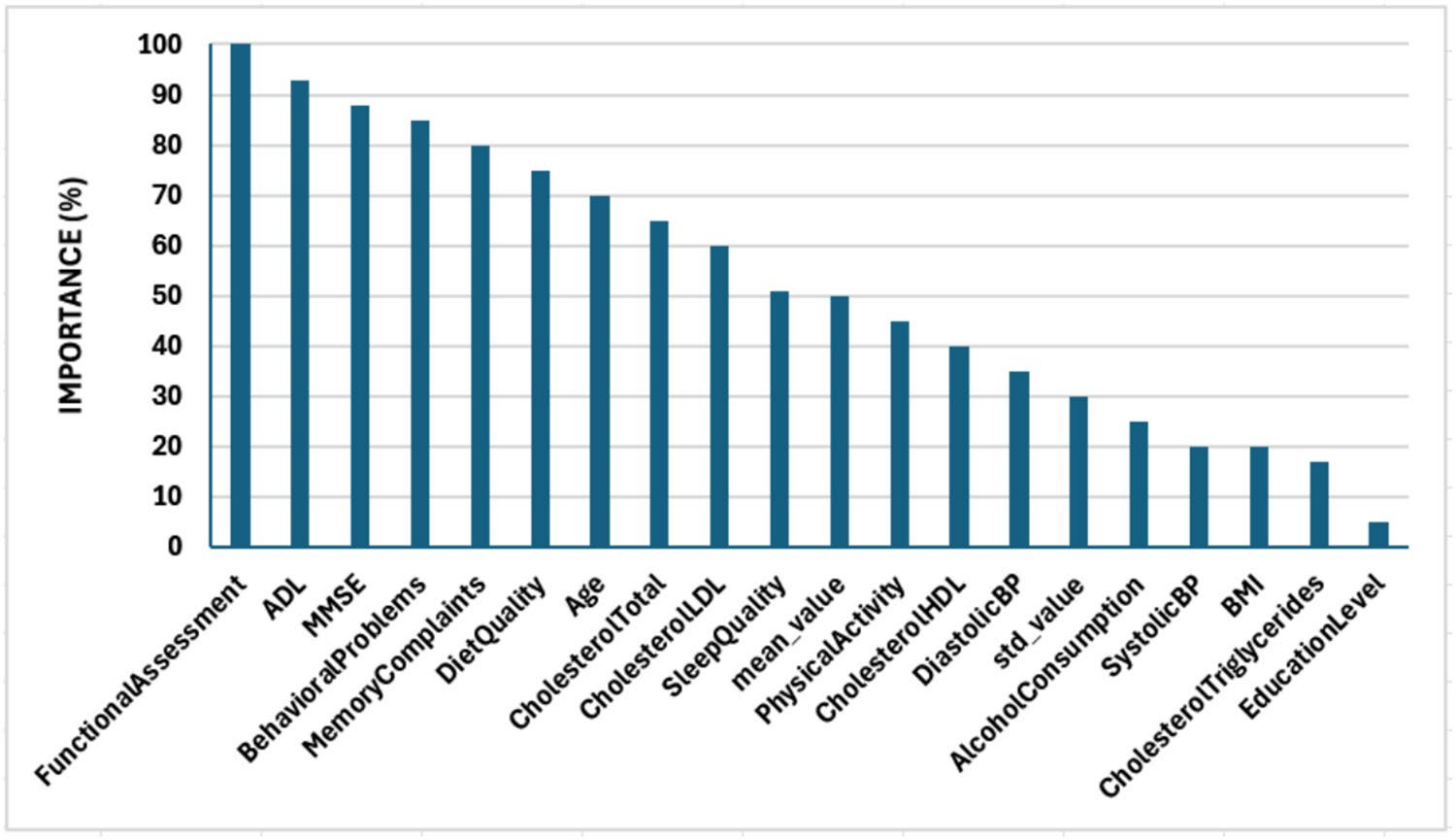
Top 20 clinical features ranked based on their importance for Alzheimer’s disease classification, as derived from a LightGBM model trained on the Kaggle clinical dataset.

To gain insights into which original clinical features are most influential for prediction within the clinical dataset, a full feature importance analysis was performed. While the primary classification model for Pathway1 combines a 1D CNN with a Random Forest, the CNN component transforms the input features into a complex, learned representation. Feature importance derived from the Random Forest in this pipeline would reflect the importance of these learned features, rather than the original clinical attributes. Therefore, to directly assess the relevance of the original clinical features (such as Age, MMSE, Diet Quality, etc.), we trained a separate LightGBM model directly on the preprocessed original clinical dataset. LightGBM is a gradient boosting framework that intrinsically provides robust feature importance scores based on how much each feature contributes to reducing impurity (or loss) across the boosting rounds^24^. These scores were normalized to a 0–100 scale for easier comparison, as depicted in Fig. 7. This separate analysis allows researchers to identify which raw clinical inputs were most discriminative for AD status in this dataset, providing valuable interpretability alongside the performance of the main Pathway1 model.

The analysis highlighted Functional Assessment and ADL (Activities of Daily Living) scores as the most important features, followed closely by MMSE (Mini-Mental State Examination) and behavioral problems. This aligns well with clinical understanding, as these features directly measure cognitive and functional decline symptomatic of AD. Lifestyle factors like Diet Quality and Sleep Quality also appeared significant. Age, a known major risk factor, also showed high importance. Medical history items like Family History of AD and cardiovascular disease were moderately important. Features like Education Level and Cholesterol Triglycerides had relatively lower importance scores in this dataset. This analysis supports the model’s reliance on clinically relevant indicators and provides valuable interpretability for predictions made using Pathway1.

### Key brain regions in Alzheimer’s classification

Furthermore, to understand which regions of the MRI scans contribute most to the VGG19 model’s predictions in Pathway2, a Class Activation Mapping (CAM)^25^ was utilized. CAM helps visualize the regions in the input image that were most influential for a specific class prediction by weighting the activation maps of the last convolutional layer. This technique requires a Global Average Pooling (GAP) layer before the final classification layer, which was incorporated from the VGG19 architecture for visualization purposes.

**Figure 8:**
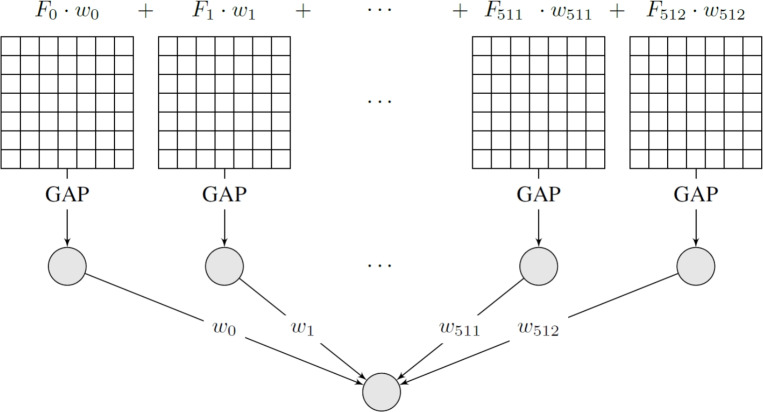
Conceptual illustration of Class Activation Mapping (CAM). Weighted summation of feature maps from the last convolutional layer after Global Average Pooling highlights regions influential for the predicted class.

Technically, the CAM for a given class *k* at a spatial location (*x*, *y*) is computed as a weighted sum of the activation maps $F_i$ from the last convolutional layer:

8 $$\begin{aligned} C_k(x, y) = \sum _{i = 1}^{N_{features}}{w_i^k F_i(x, y)} \end{aligned}$$

where $N_{features}$ is the number of feature maps (i.e., 512 for VGG19), and $w_i^k$ is the weight connecting the *i*-th feature map (specifically, its value after GAP) to the $k^{th}$ class output node in the final dense layer. The resulting map $C_k(x, y)$ highlights spatial regions that are highly predictive of class *k*. This process is illustrated conceptually in Fig. 8.

**Figure 9:**
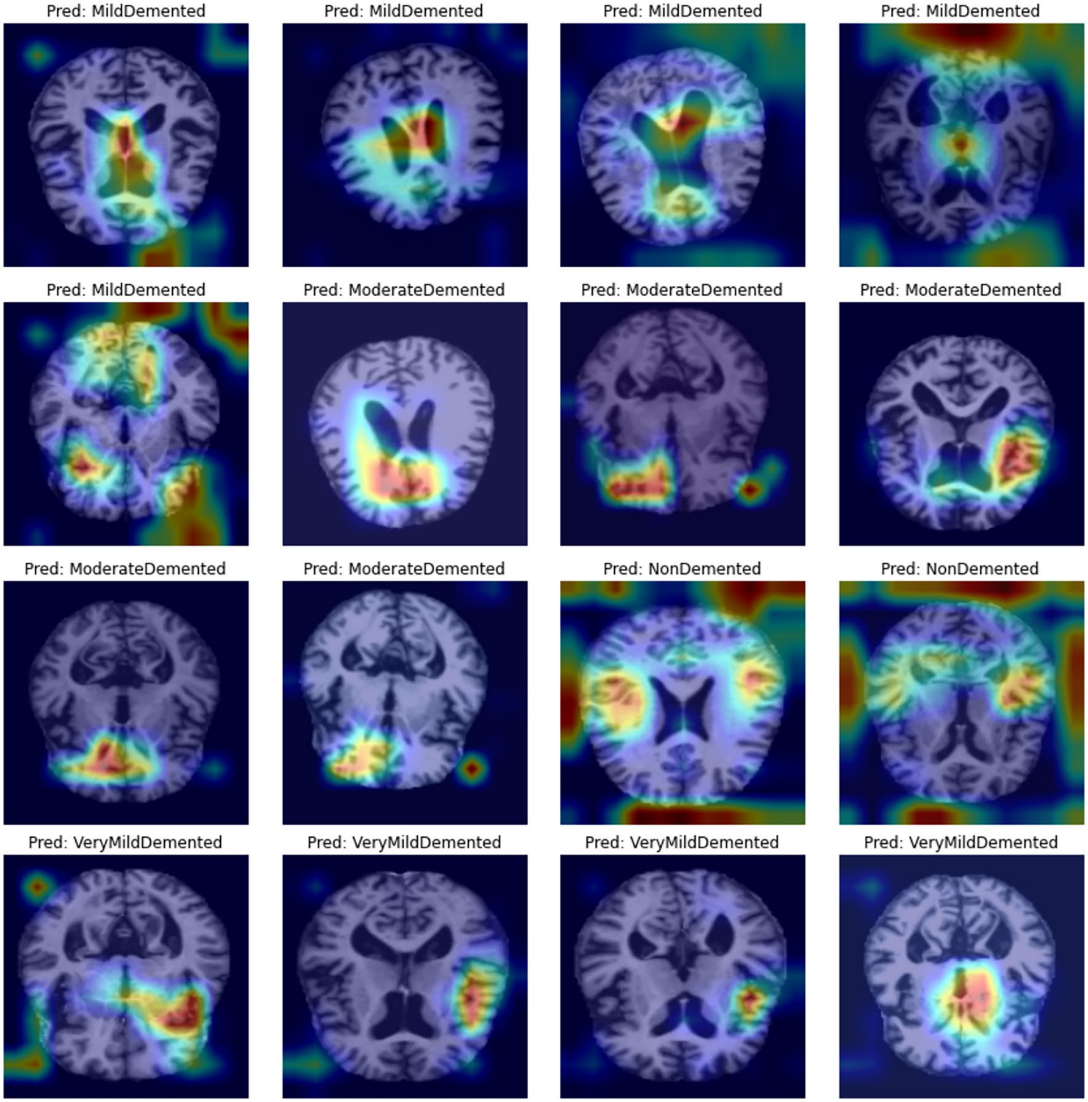
Sample MRI brain images overlaid with CAM heatmaps. Red/Yellow regions indicate high activation (most influential for classification), Green moderate, and Blue low activation.

Figure 9 shows sample MRI brain images from the Kaggle dataset overlaid with CAM heatmaps generated for their predicted classes. These heatmaps visually demonstrate that Pathway2’s model focuses on regions known to be associated with AD pathology, such as areas within the temporal lobe, including the hippocampus, and certain cortical structures. While the resolution of CAM is limited by the downsampling in the CNN, this interpretability measure confirms that the deep learning model is attending to medically relevant areas in the images when making its classification decisions, enhancing trust in its predictions.

The CAM visualizations presented in Fig. 9 offer helpful details about the brain regions most influential to the VGG19 model’s classification decisions across different stages of dementia. Notably, the highlighted areas consistently include regions within the temporal lobe particularly around the hippocampus and parahippocampal gyrus which are well-established as early sites of neurodegeneration in Alzheimer’s disease^26^. For cases classified as ”MildDemented” and ”ModerateDemented,” the model often focuses on bilateral medial temporal structures and adjacent cortical areas, suggesting its sensitivity to anatomical changes associated with memory and cognitive decline. In contrast, the ”NonDemented” and ”VeryMildDemented” predictions exhibit more diffuse or peripheral activations, indicating either a lack of pronounced pathological features or early, subtle degeneration. While the spatial resolution is constrained by the model’s architecture, these CAM overlays align with known neuropathological patterns, supporting the clinical relevance of the deep learning approach and enhancing interpretability of its predictions.

### Comparison with baselines

Table 4 presents a comparison of the performance of the proposed DeepALZNET pathways with selected benchmark studies using various methods and datasets, including ADNI and OASIS. The primary evaluations were conducted on the Kaggle datasets as detailed in the previous sections. For comparison with existing work, we also conducted preliminary evaluations of DeepALZNET Pathway1 and Pathway2 on subsets of the ADNI and OASIS datasets. Specifically, for the MRI-based Pathway2, we utilized 3k T1-weighted MRI images from the ADNI dataset and 1k images from the OASIS-1 and OASIS-3 collections, all preprocessed using skull stripping, intensity normalization, and resizing to $224\times 224$ pixels. Images were categorized into four classes (NonDemented, VeryMildDemented, MildDemented, ModerateDemented) based on corresponding clinical annotations. The class distributions were approximately balanced by undersampling the majority classes. A segmented 70/30 train/test split were employed and applied data augmentation during training. For the clinical-based Pathway1, we extracted structured features for 1.2k subjects from ADNI and 850 from OASIS, selecting only those with complete cognitive, behavioral, and demographic records. Feature engineering followed the same procedure as in the Kaggle-based experiment, and missing values were handled by mean imputation. Models were trained using the same CNN+Random Forest architecture and its hyperparameters. The reported results in Table 4 reflect an average performance across three independent runs.

**Table 4:** Comparison of DeepALZNET performance with recent baseline methods for AD classification.

Method	Data modality	Dataset (s)	Accuracy (%)
Clinical data based classification			
**DeepALZNET 1**	Clinical	Clinical^32^	**90.2**
**DeepALZNET 1**	Clinical	ADNI	**94**
**DeepALZNET 1**	Clinical	OASIS	**93**
Multi-level stacking (ML)^11^	Clinical	ADNI	92.08
Random forest^8^	Clinical	OASIS	86.3
Naive Bayes^18^	Clinical	ADNI	82
AdaBoost^18^	Clinical	ADNI	77.1
MRI based classification			
**DeepALZNET 2**	MRI	MRI^33^	**92.2**
**DeepALZNET 2**	MRI	ADNI	**98.5**
**DeepALZNET 2**	MRI	OASIS	**98.1**
CNN+SVM^22^	MRI	OASIS	86.2
CNN^17^	MRI	OASIS	98.2
U-Net^17^	MRI	ADNI	92.45
VGGNet 3D^17^	MRI	ADNI	88.8
BrainNet2D^19^	MRI	OASIS	88
Naive Bayes^23^	MRI	ADNI	78
ResNet152^18^	MRI	ADNI	99.2
ResNet152^18^	MRI	OASIS	97.1
InceptionV3^18^	MRI	OASIS	98.8

The results presented in Table 4 suggest that DeepALZNET performs competitively across different datasets and modalities when compared to various baseline methods. The clinical pathway (DeepALZNET 1) shows strong performance on the Kaggle dataset (90.2%) and promising accuracies on subsets of ADNI (94%) and OASIS (93%) compared to other clinical-based methods. Similarly, the MRI pathway (DeepALZNET 2) achieves high accuracy on the Kaggle MRI dataset (92.2%) and competitive results on subsets of ADNI (98.5%) and OASIS (98.1%) compared to other MRI-based approaches. While direct quantitative comparison with published results on the full ADNI/OASIS datasets is subject to variations in preprocessing, specific cohorts used, and evaluation protocols, these preliminary results on subsets indicate the potential generalizability and effectiveness of the DeepALZNET framework.

## Conclusion

This study introduced DeepALZNET , demonstrating the potential of dedicated deep learning pathways for Alzheimer’s disease detection using clinical records or MRI data. The framework’s hybrid CNN-Random Forest model for clinical analysis and the VGG19-based network for neuroimaging both yielded strong predictive performance. Evaluations confirmed high diagnostic accuracy for both approaches, exceeding 90%, highlighting the efficacy of these specialized strategies in capturing disease patterns within distinct data modalities. The incorporation of interpretability techniques like CAM further enhance clinical relevance by visualizing model decision-making.

However, challenges remain, including dataset limitations, class imbalance, and the interpretability of deep learning models in clinical decision-making. Future work will focus on improving model explainability and leveraging datasets that combine clinical and MRI data from the same patients. This integration would allow the model to learn complementary features, leading to better classification performance and a more comprehensive diagnostic approach.

## Data Availability

The data is fully available for public use and was fully cited in the manuscript as follows: Kaggle MRI dataset^33^ : https://www.kaggle.com/datasets/uraninjo/augmented-alzheimer-mri-dataset. Kaggle Clinical dataset^32^ : https://doi.org/10.34740/kaggle/dsv/8668279. ADNI dataset^12^ : https://adni.loni.usc.edu/data-samples/adni-data/. OASIS dataset^9,10^ : https://sites.wustl.edu/oasisbrains/.
